# Contrasting patterns of insecticide resistance and knockdown resistance (*kdr*) in the dengue vectors *Aedes aegypti* and *Aedes albopictus* from Malaysia

**DOI:** 10.1186/s13071-015-0797-2

**Published:** 2015-03-25

**Authors:** Intan H Ishak, Zairi Jaal, Hilary Ranson, Charles S Wondji

**Affiliations:** Department of Vector Biology, Liverpool School of Tropical Medicine, Pembroke Place, Liverpool, L3 5QA United Kingdom; School of Biological Sciences, Universiti Sains Malaysia, 11800 Penang, Malaysia

**Keywords:** Dengue, *Aedes aegypti*, *Aedes albopictus*, Insecticide resistance, Knockdown resistance

## Abstract

**Background:**

Knowledge on the extent, distribution and mechanisms of insecticide resistance is essential for successful insecticide-based dengue control interventions. Here, we report an extensive resistance profiling of the dengue vectors *Aedes aegypti* and *Aedes albopictus* across Malaysia and establish the contribution of knockdown resistance mechanism revealing significant contrast between both species.

**Methods:**

*Aedes* mosquitoes were collected from four states in Malaysia in 2010 using ovitraps and tested against six major insecticides using WHO bioassays. Knockdown resistance (*kdr*) was investigated in both species.

**Results:**

A moderate resistance to temephos was detected from samples collected in 2010 in Penang, Kuala Lumpur, Johor Bharu and Kota Bharu (1.5 < RR < 3.3). A widespread and multiple resistances was observed in *Ae. aegypti* particularly against pyrethroids, DDT and bendiocarb. Mosquitoes from Kuala Lumpur consistently had the highest resistance levels and was the only population showing a moderate resistance to malathion (91% mortality). The resistance profile of *Ae. albopictus* contrasted to *Ae. aegypti* with full susceptibility to pyrethroids except in Kuala Lumpur where moderate resistance is observed. PBO synergist assays suggest metabolic resistance mechanisms play a major role in resistance in both species. Two kdr mutations, F1534C and V1016G, were detected in *Ae. aegypti* across Malaysia but neither of these mutations were found in *Ae. albopictus*. Additionally, signatures of selection were detected on the Voltage-gated sodium channel gene in *Ae. aegypti* but not in *Ae. albopictus*. The presence of the 1534C allele was significantly associated with pyrethroid resistance and an additive effect to pyrethroid resistance was observed in individuals containing both kdr alleles.

**Conclusions:**

Findings from this study will help to design and implement successful insecticide-based interventions against *Ae. aegypti* and *Ae. albopictus* to improve dengue control across Malaysia.

**Electronic supplementary material:**

The online version of this article (doi:10.1186/s13071-015-0797-2) contains supplementary material, which is available to authorized users.

## Background

Dengue is the most rapidly spreading vector-borne disease with approximately 50 million cases of infection worldwide [1,2]. Malaysia is one of the most affected countries in Southeast Asia with 46,171 cases reported in 2010 with 134 deaths [3]. The main dengue vectors *Aedes aegypti* and *Ae. albopictus are* widely distributed throughout Malaysia [4,5]. These two species overlap in their geographical distribution although *Ae. aegypti* is preferentially found in rapidly developing areas with less vegetation whereas *Ae. albopictus* prefers conditions with more vegetation and is generally more exophlic than *Ae. aegypti* [6].

The main dengue vector control methods in Malaysia are adulticiding using permethrin, deltamethrin and malathion and larviciding with temephos and *Bacillus thuringiensis israelensis (Bti)* [3]. Insecticides are widely used in Malaysia not only by the Ministry of Health (MoH) operators but also by private companies and the community to control mosquitoes as well as other household pests [7]. Such intense use of insecticides is one of the main causes of increasing reports of insecticide resistance in *Aedes* populations in Malaysia and throughout the world [8] threatening the continued success of current vector control interventions. In Malaysia, evidence of resistance towards permethrin and temephos has been recorded in both *Aedes* species in Kuala Lumpur and Penang [9,10]. However, the susceptibility profile against other insecticide classes remains unknown. In addition, the geographical distribution and the extent of insecticide resistance in *Ae. aegypti* and *Ae. albopictus* populations across Malaysia remain to be established. Such information is needed in order to design and implement suitable control interventions against these species.

The two major causes of insecticide resistance are alterations in the target sites and increase in the rate of insecticide metabolism [11]. While metabolic resistance is caused primarily by three enzyme families, the cytochrome P450s, the esterases and glutathione S-transferases (GSTs), target site resistance is conferred by one or several mutations in the insecticide target site [11]. One of the main target site mutations is the ‘knockdown resistance’ mutation (*kdr*) conferring resistance to pyrethroid and DDT insecticides [11]. Mutations at three codon positions of the Voltage-Gated Sodium Channel (VGSC) gene (I1011M/V, V1016G/I and F1534C) have been primarily associated with both pyrethroids and DDT resistance in various *Ae. aegypti* populations [12-14]. Additional mutations such as S989P have also been associated with pyrethroid resistance in *Ae. aegypti* [15]. Some of these mutations such as the F1534C mutation have been reported in countries neighbouring Malaysia such as in Thailand [16] and Vietnam [17]. The F1534C mutation has also been reported in *Ae. albopictus* from Singapore [18]. Nothing has been reported on the presence of these target site mutations in *Aedes* mosquitoes in Malaysia.

Here, we present an extensive resistance profiling for all insecticide classes in Malaysian *Ae. aegypti* and *Ae. albopictus* populations across a South/North transect. The contribution of both knockdown resistance (*kdr*) and metabolic resistance mechanisms is also characterised providing key information necessary for the implementation of suitable evidence-based control strategies against both *Aedes* species to help reduce dengue burden in Malaysia.

## Methods

### Mosquito samples

*Ae. aegypti* and *Ae. albopictus* mosquitoes were collected in July and August 2010 across Malaysia. Approximately 80 ovitraps were set up in four states; Penang (PG) (Northwest), Kota Bharu (KB) (Northeast), Kuala Lumpur (KL) (Centre) and Johor Bharu (JB) (South). The collection sites were geographically spread out but with a focus on residential areas that were notorious dengue transmission hotspots and regularly sprayed with insecticides notably permethrin and malathion using thermo fogging by the Health Ministry. The traps were collected five days later. Larvae were also collected from old tyres, flower pots, tree holes and containers that held water.

### Mosquito rearing

Egg and larval collections from all four locations were brought to the Vector Control Research Unit (VCRU) in Penang where larvae were fed with larval food containing grounded dog biscuit, beef liver, powdered milk and yeast with a ratio of 2:1:1:1. After emergence, adult *Aedes* mosquitoes were morphologically identified to species based on the pattern on the thorax and put into two separate cages, fed with 10% sucrose solution and were later given a blood meal to induce egg laying. Egg papers were dried at room temperature and kept in a sealed plastic bag. Both the egg papers and dead mosquitoes kept in silica gel were brought back to Liverpool School of Tropical Medicine (LSTM) under the LSTM import license from DEFRA. The egg batches were then hatched in the insectary in water supplemented with hay infusion solution. Larvae were reared as above and the adults were given 10% sucrose solution and kept at a room temperature of 27 ± 2°C with relative humidity of 70 ± 10%.

### Insecticide susceptibility tests

#### Larval bioassays

The larval bioassays were conducted according to WHO guidelines [19] using F_2_ generation larvae. 1 ml of temephos insecticide (1 g/L of original concentration) (Sigma Aldrich) was diluted with ethanol and mixed with 249 ml distilled water. Four replicates of 10 different concentrations between 0.002 ppm to 0.075 ppm and ethanol only as control were tested on 25 late third instar to early fourth instar larvae. The mortality was recorded after 24 hours of exposure. Larvae that were unable to swim up to the surface were counted as dead and the larvae that have pupated were omitted from the final total. The lethal concentration that kills 50% of the tested samples (LC50) was calculated using probit analysis (PASW statistics 18 software). Resistance ratios (RR) were calculated by comparing LC50s with data obtained from the New Orleans susceptible strain of *Ae. aegypti* and an *Ae. albopictus* strain from the Malaysia Vector Control Research Unit (VCRU).

#### Adult insecticide bioassays

Bioassays were carried out according to WHO protocol [20] using 2–5 day-old F_2_ generation of *Ae. aegypti* and *Ae. albopictus* mosquitoes with 4 replicates of 25 mosquitoes per tube. The insecticides that were tested are: 0.75% Permethrin (Type I pyrethroid), 0.05% Deltamethrin (Type II pyrethroid), 4% DDT (organochlorine), 4% Dieldrin (organochlorine), 0.1% Bendiocarb (Carbamate) and 5% Malathion (organophosphate). Survivors after the bioassays were stored at −80°C freezer whereas dead mosquitoes were kept in silica gel Eppendorf tubes. Insecticide papers were provided by University of Sains Malaysia.

#### Synergist assays with PBO

The effect of pre-exposure to the synergist, piperonyl butoxide (PBO) was also assessed to investigate the potential role of oxidase-specific metabolic resistance mechanisms. Adult 2–5 days old mosquitoes were exposed to papers impregnated with 4% PBO for one hour and then immediately exposed to four insecticides; permethrin, deltamethrin, DDT or bendiocarb using WHO susceptibility test kits. Mortality was scored after 24 hours and compared to the results obtained with each insecticide without PBO exposure and to a control sample exposed only to PBO.

### Investigation of knockdown resistance (*kdr*)

#### Search for potential *kdr* mutations in both species

To identify potential *kdr* mutations, a fragment of the coding region of the VGSC gene spanning exon 19 to exon 31 (covering the 989, 1011, 1016 and 1534 coding positions) was amplified from cDNA samples and directly sequenced. RNA was extracted from pools of three batches of 10 mosquitoes (not exposed to any insecticide for *Ae. aegypti* or from DDT resistant for *Ae. albopictus*) from all the four locations using Picopure kit (Arcturus). cDNA were synthesised using the Superscript III kit (Invitrogen) with oligo-dT20 and RNase H as previously described [21,22]. The PCR was carried out using 10 pmol of each primers (Additional file 1: Table S1) and 20 ng of cDNA as template in 15 μl reactions containing 1X HF buffer A, 0.2 mM dNTPs, 1.5 mM MgCl_2_, 1U Phusion Taq. The cycle conditions were 98°C for 1 min and 35 cycles of 98°C for 10 s, 63°C (60 for *Ae. albopictus*) for 30 s and 72°C for 1 min and 30 s, followed by a final extension step of 72°C for 10 min. The samples were purified using the Qiaquick PCR purification kit (Qiagen) and sequenced directly. The sequences were aligned and analysed as indicated above.

### Genotyping of *kdr* mutations in *Aedes aegypti*

#### Development of pyrosequencing assays

Genomic DNA was extracted using the Livak method [23]. The presence of the three *kdr* mutations known in *Ae. aegypti* [I1011V (or M)] [12], [V1016I (or G)] [14] and [F1534C] [13] was assessed by genotyping 30 F_0_ females from all four populations using the pyrosequencing method. Subsequently, the potential role of these *kdr* mutations in the resistance to pyrethroids or DDT was assessed by establishing the correlation between genotypes and resistance phenotype using 25 dead and 25 alive mosquitoes from each population after exposure to permethrin, deltamethrin and DDT by estimating the odds ratios and the statistical significance based on the Fisher exact probability test.

The pyrosequencing assay was performed as previously described by Wondji et al. [24]**.** Briefly, a PCR amplification of the genomic fragment to sequence was first carried out using 10pmol of each primer pair (Additional file 1: Table S2) to genotype the three different *Kdr* mutations; *Kdr*1011, *Kdr*1016 and *Kdr*1534 in a final reaction volume of 15 μl containing 1X HotStar Taq buffer, 0.2 mM dNTPs, 1.5 mM MgCl_2_, 1U HotStar Taq and 20 ng gDNA. The PCR parameters were 95°C for 15 minutes and 50 cycles of 94°C for 20 seconds, 55°C for 30 seconds and 72°C for 30 seconds, followed by an extension step of 72°C for 5 minutes. The PCR products were used for the pyrosequencing assay as previously described [24]. Attempts were also made to genotype these three mutations in *Ae. albopictus.*

#### V1016G genotyping using allele specific PCR in *Ae. aegypti*

The 1016 *kdr* mutation was genotyped using the allele specific PCR method as previously described by Saavedra-Rodriguez et al. [14] as the pyrosequencing consistently failed to detect it probably because of the presence of two consecutive alternative mutations*.* This mutation was genotyped in all alive and dead mosquitoes for deltamethrin, permethrin and DDT insecticides to assess its correlation with resistance to these insecticides. PCR was performed in a 25 μl volume in 96-well plates (Agilent technologies) containing 12.5 μl of Brilliant III Ultra-Fast SYBR Green QPCR Master Mix (Agilent) 25 pmoles of each primer, 100 ng of template DNA using the MX3005 qPCR system (Agilent Technologies). Thermal cycling conditions were: 95°C for 12 min; 39 cycles of 95°C for 20 s; 60°C for 1 min; 72°C for 30 s; 72°C for 5 min (final extension) and ramp from 65°C to 95°C at a rate of 0.2°C/s (melting curve).

#### Polymorphism of the voltage-gated sodium channel (VGSC) gene in *Ae. aegypti*

To assess the correlation between the polymorphism of the VGSC gene and resistance, and to detect possible signatures of selection, a fragment of this gene spanning the F1534C mutation (intron 26 to exon 29) was amplified and sequenced in five permethrin resistant (alive) and five susceptible (dead) mosquitoes after exposure to permethrin from PG, KL, JB and KB. PCR reactions were carried out using 10 pmol of each primer (Additional file 1: Table S1) and 20 ng of genomic DNA as template in 15 μl reactions containing 1X Kapa Taq buffer, 0.2 mM dNTPs, 1.5 mM MgCl_2_, 1U Kapa Taq (Kapa biosystems). The cycle conditions were 95°C for 5 min and 35 cycles of 94°C for 30 s, 57°C for 30 s and 72°C for 1 min, followed by a final extension step of 72°C for 10 min. The samples were purified using the Qiaquick PCR purification kit (Qiagen) and sequenced directly (Macrogen, Korea). The sequences were aligned using ClustalW [25]. DnaSP v5.10 [26] was used to define the haplotype phase and the genetic parameters including nucleotide diversity π, haplotype diversity and the D and D* selection estimates. A maximum likelihood tree of the haplotypes was constructed using MEGA 5.2 [27] whereas a haplotype network was built using the TCS program [28] to further assess the potential connection between haplotypes and resistance phenotypes.

## Results

### Resistance profiling to insecticides

#### Larval bioassay for temephos

For *Ae. aegypti*, the Penang strain exhibited the highest LC_50_ (0.008 ppm) but with only a moderate resistance ratio (RR) of 2 when compared to the susceptible NO strain while the RR for both Kuala Lumpur and Johor Bharu were 1.5 (Table 1). Slightly higher LC_50_ were observed in *Ae. albopictus*, with the highest recorded in Penang (0.02 ppm) with RR of 3.3 (Table 1).

**Table 1:** **Temephos LC**_**50**_ **and RR of** ***Ae. aegypti and Ae. albopictus*** **Malaysian strains against susceptible laboratory strains**

**Strain**	**Sample size**	**LC**_**50**_ **,ppm (95%** **C.I.)**	**RR**
*Ae. aegypti*			
New Orleans	640	0.004 (0.003 – 0.006)^a^	1
Penang	640	0.008 (0.008 – 0.009)^a^	2
Kuala Lumpur	640	0.006 (0.005 – 0.006)	1.5
Johor Bharu	640	0.006 (0.005 – 0.006)	1.5
Kota Bharu	N/A	-	
*Ae. albopictus*			
VCRU	640	0.006 (0.006 – 0.007)	1
Penang	640	0.020 (0.018 – 0.021)^a^	3.3
Kuala Lumpur	640	0.015 (0.014 – 0.016)^a^	2.5
Johor Bharu	N/A	-	-
Kota Bharu	N/A	-	-

Four replicates tested for each temephos concentration.C.I.: Confidence Interval. ^a^Statistically significant.

### Adult bioassays

#### *Ae. aegypti* resistance pattern

**Figure 1:**
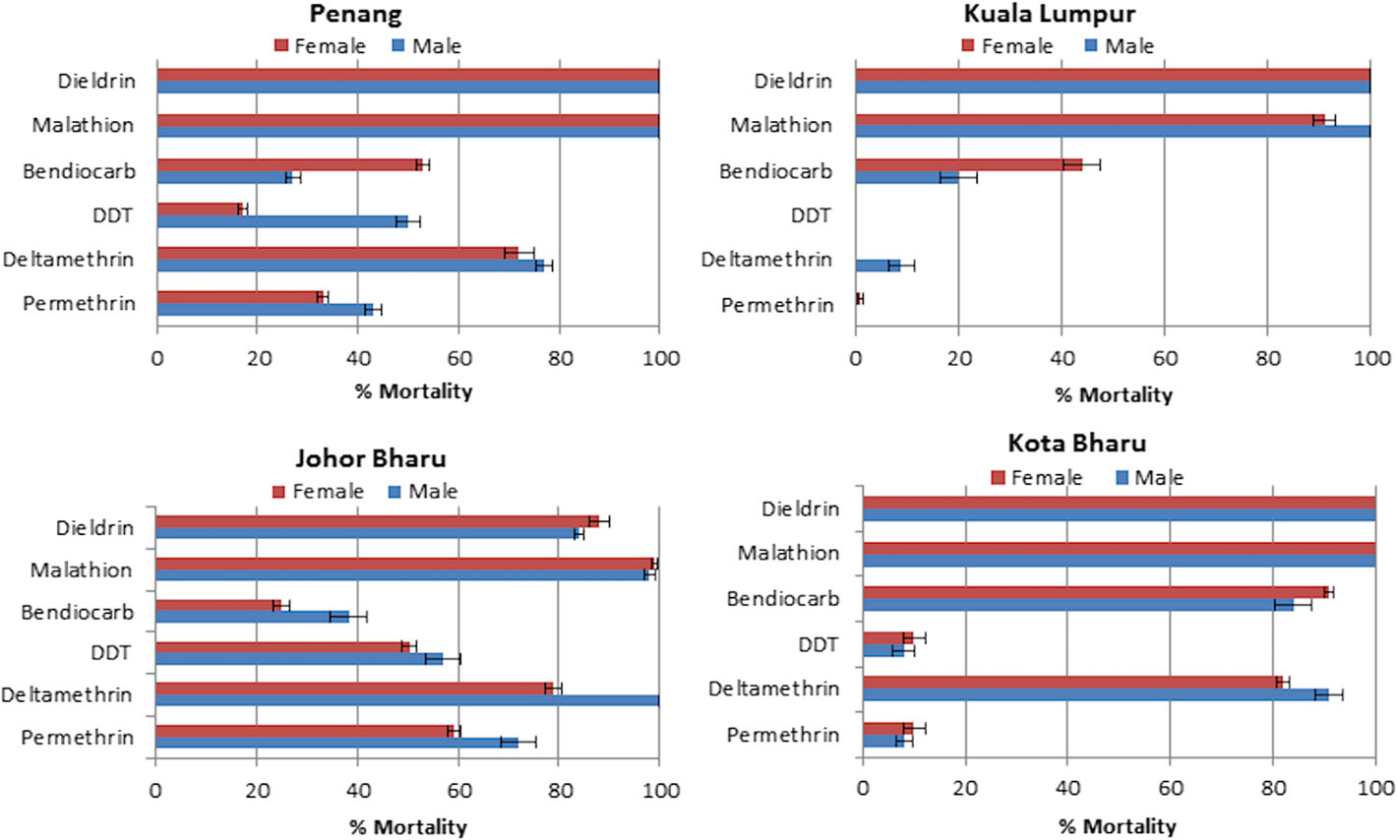
**Resistance profiles to different insecticide classes in** ***Ae. aegypti*** **populations across Malaysia.** Error bars represent standard deviation (n = 4).

Because diagnostic doses for WHO adult bioassays have not yet been defined for most insecticides for *Ae. aegypti* and *Ae. albopictus*, *Anopheles* mosquitoes’ diagnostic doses were used in this study. These doses are higher than in *Aedes* for the few doses defined in 1992 [29]. Despite these higher diagnostic doses, resistance was observed to both Type I (permethrin) and Type II (deltamethrin) pyrethroids across Malaysia (Figure 1). All populations were resistant to permethrin (defined by WHO as < 90% mortality [30]) and females of all populations were also resistant to deltamethrin. The highest resistance levels to both insecticides were observed in Kuala Lumpur with nearly all mosquitoes surviving the 1 h exposure. However, in Kota Bharu, the high permethrin resistance (10% mortality) contrasted with only a moderate resistance to deltamethrin (82% mortality) (Additional file 1: Table S3; Figure 1).

All four populations were also resistant to DDT with the highest resistance level recorded again in Kuala Lumpur with no mortality after 1 h exposure (Additional file 1: Table S3; Figure 1). Widespread resistance is also observed against the carbamate bendiocarb except in Kota Bharu where 91% mortality was observed in females (Additional file 1: Table S3; Figure 1).

Full susceptibility was observed for the organophosphate malathion, except for Kuala Lumpur where a probable resistance is observed with 91% mortality (Additional file 1: Table S3; Figure 1). Similarly, a full susceptibility was observed against dieldrin except in Johru Bharu where a moderate resistance is observed with 88% mortality in females (Additional file 1: Table S3; Figure 1).

#### *Ae. albopictus* resistance pattern

**Figure 2:**
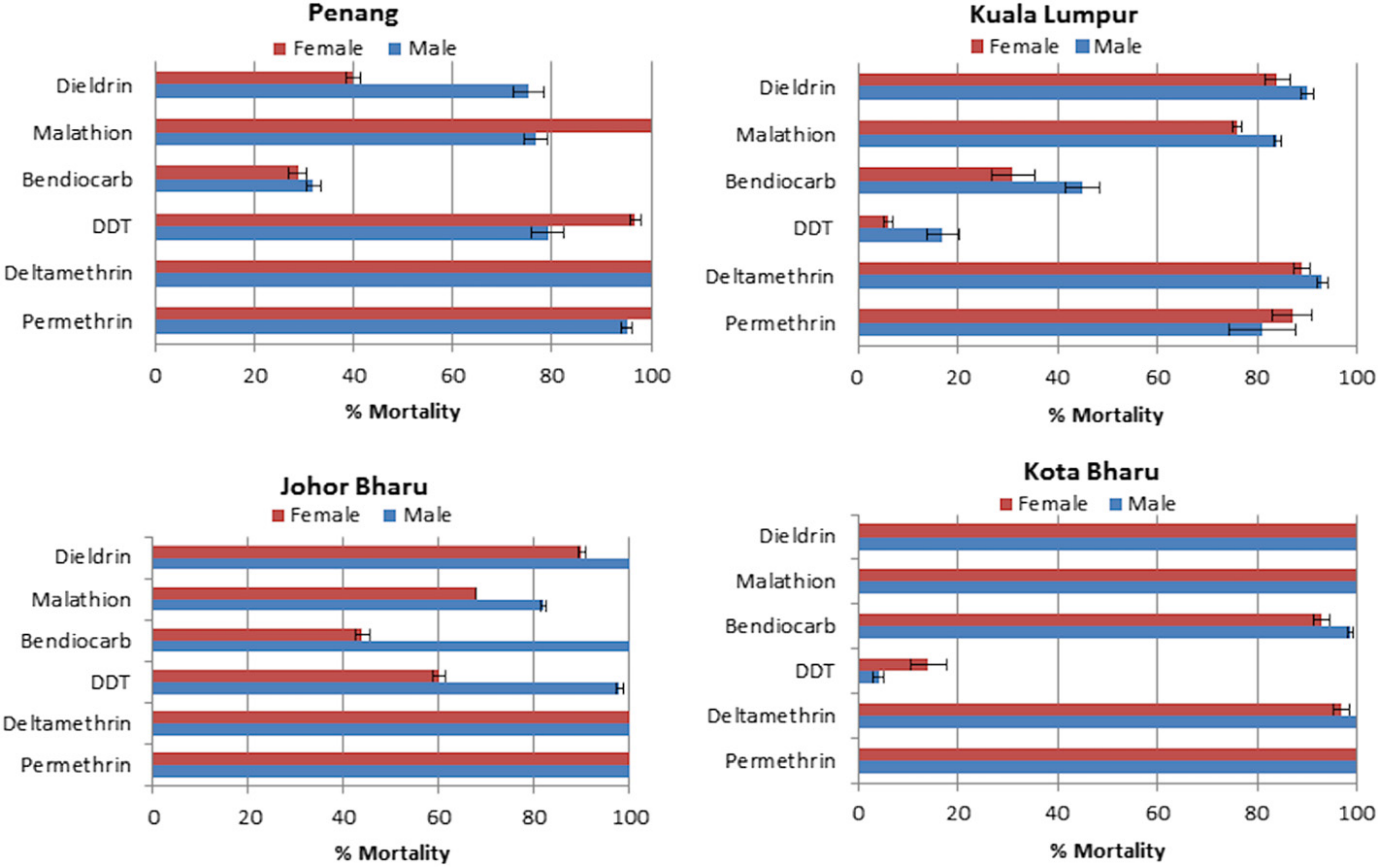
**Resistance profiles to different insecticide classes in** ***Ae. albopictus*** **populations across Malaysia.** Error bars represent standard deviation (n = 4).

In contrast to *Ae. aegypti,* populations *of Ae. albopictus* were fully susceptible to both type I and II pyrethroids except in Kuala Lumpur where a moderate resistance was observed to permethrin and to deltamethrin (87% and 89% mortality respectively) (Additional file 1: Table S4; Figure 2). A mixed resistance pattern was observed against DDT with high resistance levels recorded in Kuala Lumpur and Kota Bharu (6 and 14% mortality rate respectively), whereas a near full susceptibility is observed in Penang (96.8% mortality)(Additional file 1: Table S4; Figure 2).

High resistance levels were observed for bendiocarb in all the field strains except for Kota Bharu (93% mortality) (Additional file 1: Table S4; Figure 2). Resistance to malathion was observed in the populations of Kuala Lumpur and Johor Bharu while full susceptibility was observed in Kota Bharu (Additional file 1: Table S4; Figure 2). Resistance to dieldrin was observed in Penang, Kuala Lumpur and moderately in Johor Bharu whereas a full susceptibility is observed in Kota Bharu (Figure 2).

#### Synergist assay with PBO

##### Ae. aegypti

**Figure 3:**
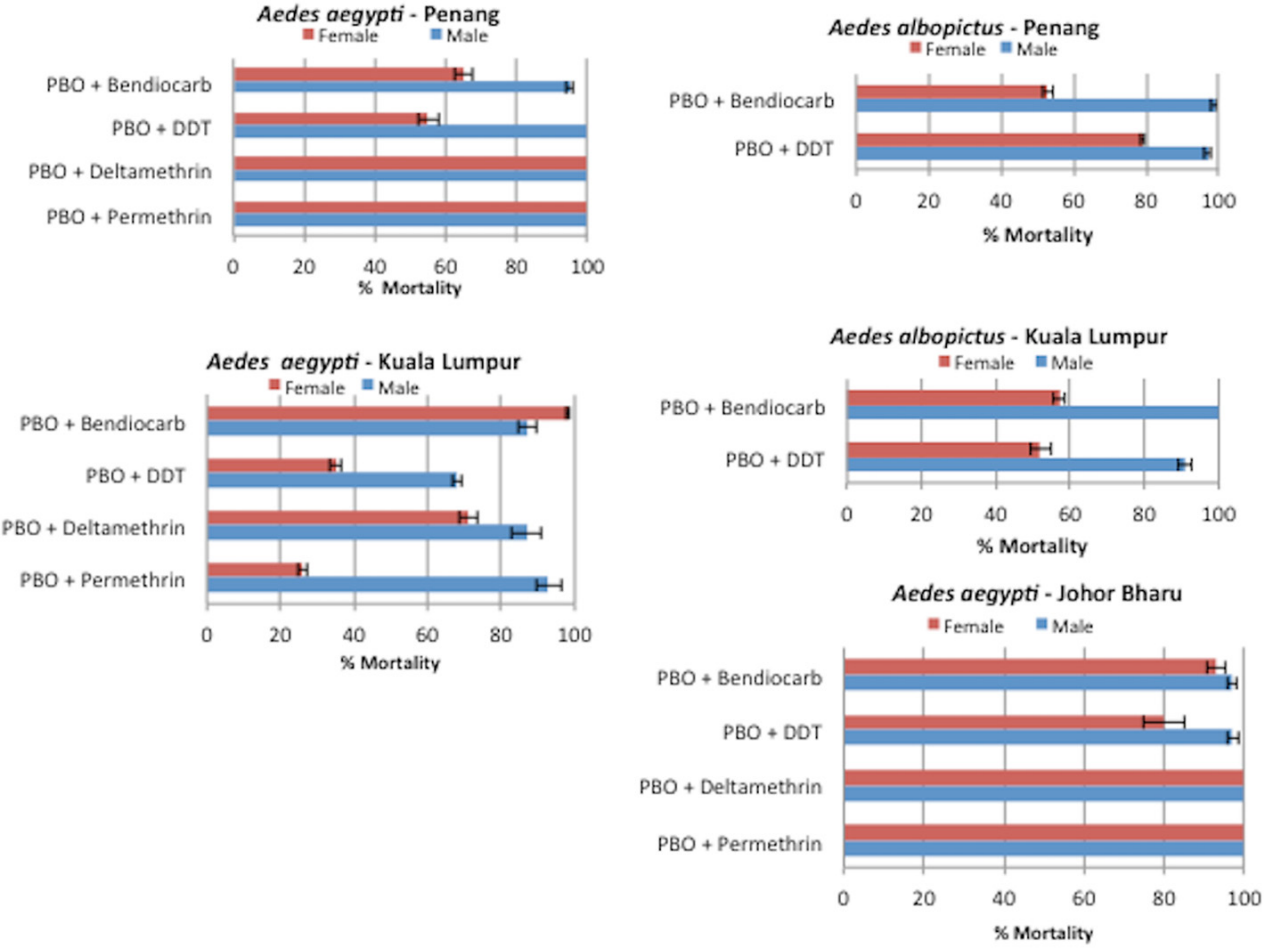
**Susceptibility profile after synergist assay with PBO.** Error bars represent standard deviation (n = 4).

A full recovery of the susceptibility (100% mortality) to both type I and II pyrethroids was observed in Penang and Johor Bharu after pre-exposure to PBO suggesting that cytochrome P450 monooxygenases are playing a predominant role in the resistance in these locations. However, only a partial recovery was observed in Kuala Lumpur with only 26% mortality in permethrin and 71% for deltamethrin for females, whereas higher recovery were observed in males (93 and 87% mortality respectively for permethrin and deltamethrin (Figure 3). Pre-exposure to PBO induced a partial recovery of susceptibility for DDT such as in Penang (55% mortality in females after PBO exposure vs 17% without PBO). However, male mosquitoes consistently exhibited a higher recovery than females such as in Penang where a full recovery (100% mortality) was observed in males after PBO. Overall, the recovery observed for DDT is lower than for pyrethroids (Additional file 1: Table S3; Figure 3). A significant recovery of susceptibility was also observed to bendiocarb in all populations tested after PBO pre-exposure. However, while this recovery was nearly total in Kuala Lumpur (98% mortality for females) and Johor Bharu (93% mortality for females), it was only moderate in Penang (53% before vs 65% after PBO pre-exposure) (Additional file 1: Table S3; Figure 3).

##### Ae. albopictus

A near full recovery of susceptibility was observed against DDT in Penang after PBO pre-exposure (99%) while in Kuala Lumpur this recovery was only partial in females (52% mortality) and nearly full in males (91% mortality). For bendiocarb, only a partial recovery of susceptibility was observed (Additional file 1: Table S4; Figure 3). This test was not performed in Kota Bharu and Johor Bharu due to limited samples.

### Genotyping of *kdr* mutations in both species across Malaysia

#### Detection of *kdr* mutations in *Ae. aegypti*

To detect potential *kdr* mutations in *Ae. aegypti* populations in Malaysia, a 2586 bp fragment spanning exon 19 to 31 was successfully amplified and sequenced in twelve cDNA samples (three for each location) from control mosquitoes non-exposed to insecticides. Because of the presence of alternative splicing, the direct sequencing and alignment generated two fragments. The first fragment covered a size of 516 bp spanning codons 989, 1011 and 1016 with 12 polymorphic sites including a single non-synonymous substitution (T-to-G) at position 1016 leading to V1016G amino acid change (GTA to GGA) in Penang, Kuala Lumpur and Kota Bharu samples (Additional file 2: Figure S1A). No mutation was detected at the 989 and 1011 positions. The second fragment covered a size of 1042 bp spanning the 1534 codon with 11 polymorphic sites including a single non-synonymous substitution at position 1534 (TCA to TGA) leading to the F1534C amino acid change in all four locations.

##### Genotyping of *kdr* mutations in *Ae. aegypti*

**Figure 4:**
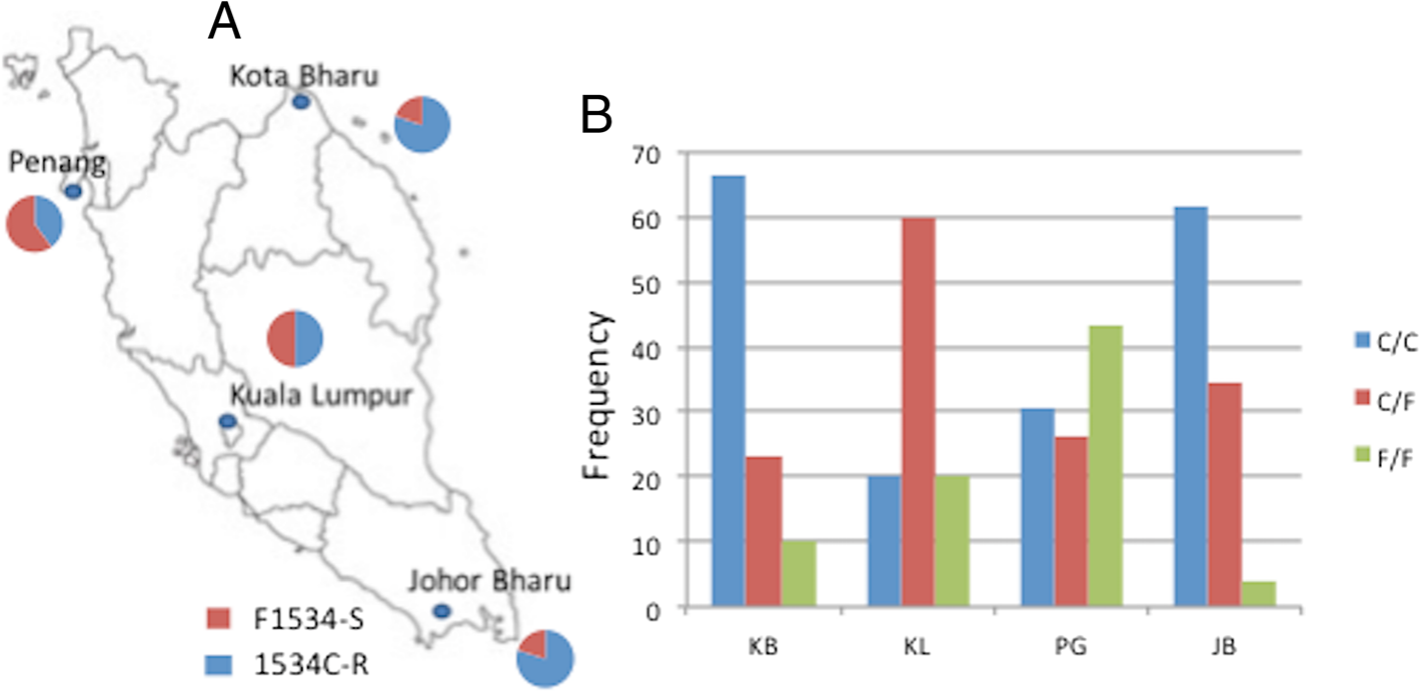
**Distribution of F1534C** ***kdr*** **mutation in field populations of** ***Ae. aegypti*** **across Malaysia. (A)** is the frequency of both alleles whereas **(B)** is the distribution of F1534C genotypes across Malaysia. R: resistant allele, S: susceptible allele.

The pyrosequencing genotyping of the two *kdr* mutations detected from cDNA sequencing at codons 1016 and 1534 in 30 F_0_ field mosquitoes from each of the four locations successfully detected the 1534C mutation but not the 1016G probably because of the presence of two consecutive polymorphisms in the sequencing regions to account for the 1016G and 1016I (Additional file 2: Figure S2). Additionally, a pyrosequencing assay of the 1011 position did not detect any mutation in the 30 F_0_ as also observed for cDNA sequencing. The frequency of the resistant 1534C allele ranged from 40% in Penang to 80% in Johor Bharu and Kota Bharu (Figure 4A). Apart from Kuala Lumpur, the genotype distribution of the F1534C mutation significantly departed from the Hardy-Weinberg Equilibrium in all other populations (P < 0.001) (Figure 4B).

##### Correlation between the F1534C genotypes and resistance phenotypes

The F1534C mutation was genotyped between resistant and susceptible mosquitoes to permethrin, deltamethrin and DDT to assess the correlation with resistance phenotypes. The 1534C resistant allele was significantly associated with permethrin and deltamethrin resistance only in Penang [odds ratio (OR) of 8.4; P = 0.018 and OR = 2.455; P = 0.027 respectively)] (Table 2; Additional file 2: Figure S3A-D). No significant correlation was observed between F1534C genotypes and DDT resistance although a high OR of 5 was observed in Johor Bharu but non-significant due to the low number of susceptible mosquitoes available.

**Table 2:** **Correlation between the 1534C resistant allele and resistance phenotypes to permethrin, deltamethrin and DDT for** ***Ae aegypti***

**Population**	**Insecticide**	**Phenotype**	**n**	**F1534C alleles**		**Odds ratio**	**P value**
				**TTC(F)**	**TGC(C)**		
Penang	Perm	R	25	32	18	8.4375	0.018
		S	8	15	1		
	Delta	R	25	18	32	2.455	0.027
		S	25	29	21		
	DDT	R	21	23	19	/	/
		S	0	0	0		
Kuala Lumpur	Perm	R	25	26	24	/	/
		S	1	0	2		
	Delta	R	25	25	25	/	/
		S	0	0	0		
	DDT	R	25	28	22	/	/
		S	0	0	0		
Johor Bharu	Perm	R	25	5	45	0.900	0.600
		S	11	2	20		
	Delta	R	25	7	43	0.84	0.49
		S	25	6	44		
	DDT	R	24	3	45	5	0.280
		S	2	1	3		
Kota Bharu	Perm	R	23	31	15	0.3871	0.091
		S	9	8	10		
	Delta	R	17	18	16	0.1212	<0.0001
		S	25	6	44		
	DDT	R	25	16	34	2.6563	0.078
		S	9	10	8		

R, Resistant; S, susceptible, /, not determined.

##### Polymorphism pattern of the VGSC fragment in *Ae. aegypti*

**Figure 5:**
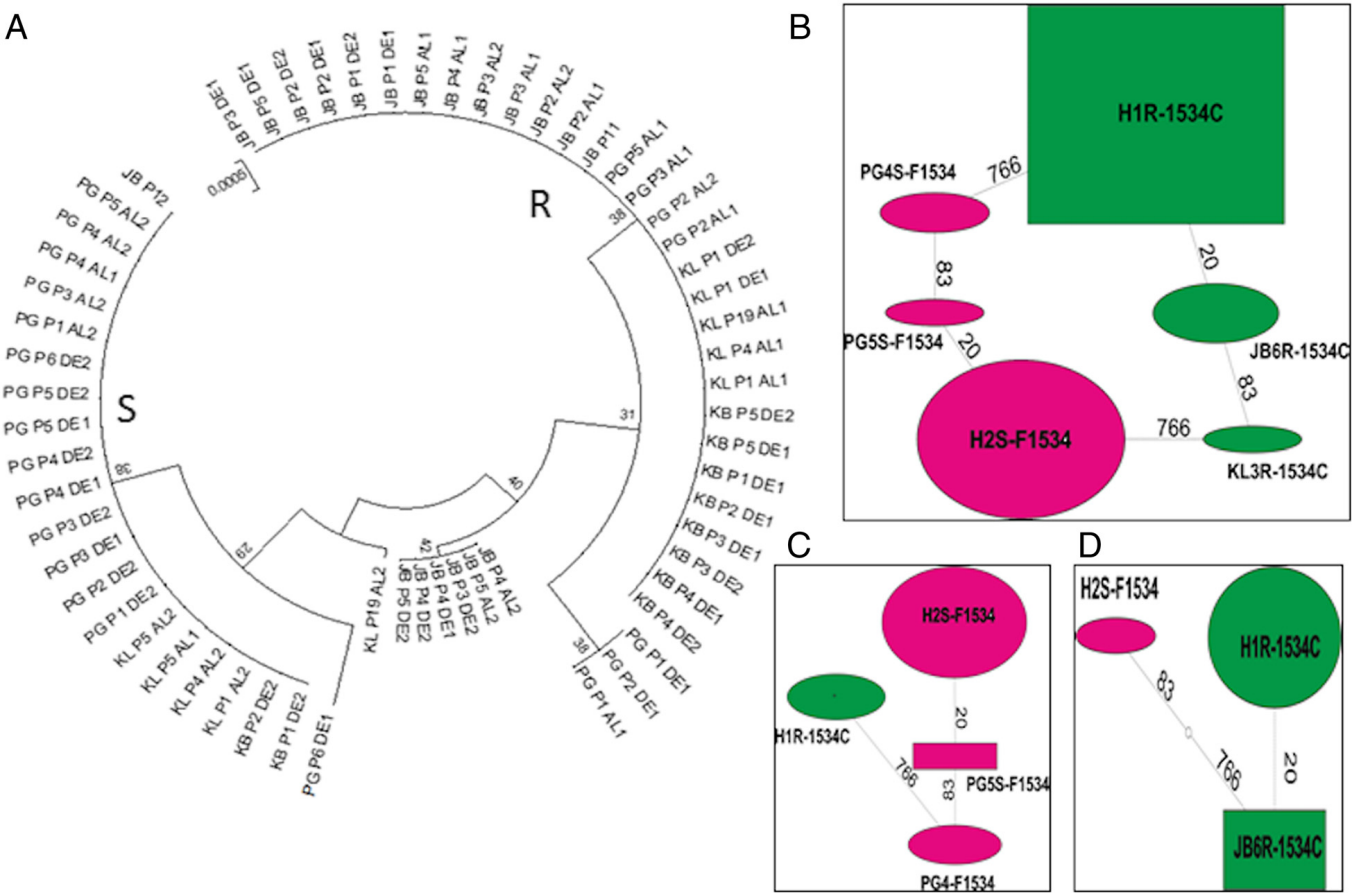
**Phylogenetic analysis of the fragment of the VGSC gene spanning the F1534C mutation in Ae. aegypti in Malaysia. (A)** Maximum likelihood phylogenetic tree of VGSC fragment showing two main clades corresponding to a resistant (R) and susceptible (S) haplotypes. **(B)** TCS network for the VGSC haplotypes between susceptible and resistant permethrin samples across whereas **(C)** and **(D)** is for Penang and Johor Bharu only respectively. Haplotypes are represented as an oval or a rectangle shape, scaled to reflect their frequencies. Lines connecting haplotypes and each node represent a single mutation event (respective polymorphic positions are given above branches). Green shapes represent haplotypes with the resistant allele (1534C); Green shapes represent haplotypes with the susceptible allele (F1534).

The polymorphism patterns of a VGSC fragment spanning the F1534C mutation (from intron 26 to exon 29) was analysed in order to assess a possible correlation between haplotypes of this gene and resistance phenotype. An 818 bp fragment was successfully sequenced and aligned in all four populations for five resistant and five susceptible mosquitoes after permethrin exposure. Overall, a low genetic diversity was observed with only 3 nucleotide substitutions observed including the 1534 position. The genetic parameters of all samples are presented in Additional file 1: Table S5. Analysis of the maximum likelihood phylogenetic tree of the VGSC sequences revealed an association between VGSC polymorphism and pyrethroid resistance as two clades corresponding to susceptible and resistant mosquitoes were observed (Figure 5A). In the total sample, 6 haplotypes were detected among which a predominant resistant haplotype H1R-1534C with a frequency of 49% and a predominant susceptible H2S-F1534 with a frequency of 30% (Figure 5B). Two other resistant haplotypes were detected, the H1R-1534C present only in Johor Bharu at 30% and the singleton KL6R-1534C haplotype present only in Kuala Lumpur. Analysis of the haplotype distribution in each location indicated a strong difference between Penang and Johor Bharu. Indeed, resistant haplotypes represent only 18% in Penang (Figure 5C) in contrast to 90% in Johor Bharu (Figure 5D). Significant positive Tajima D estimates were observed in the total sample in Penang and Johor Bharu but also in the entire sample across Malaysia indicating an excess of both low and high frequency polymorphisms in the population.

##### Genotyping of V1016G *kdr* mutation using allele specific PCR

**Figure 6:**
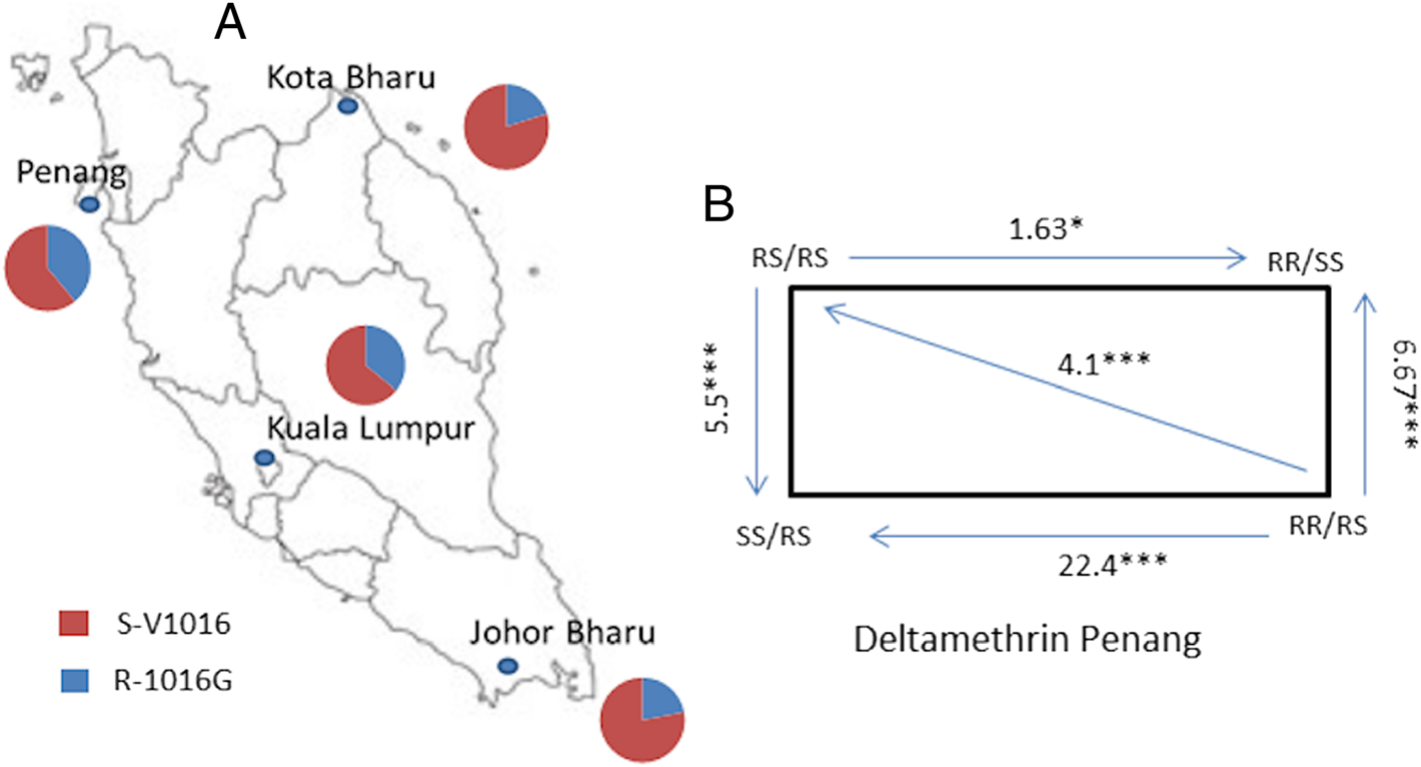
**Distribution of V1016G kdr mutation in** ***Ae. aegypti*** **across Malaysia. (A)** is the frequency of both alleles in field collected mosquitoes. **(B)** haplotypic association between the F1534C /V1016G haplotypes conferring an additive resistance. R: resistant allele, S: susceptible allele.

Since the V1016G mutation was previously undetected using the pyrosequencing method, a melting curve PCR assay described by [14] was used to genotype this mutation across Malaysia (Additional file 2: Figure S1B). The genotyping of 48 F_0_ mosquitoes from each location confirmed that the V1016G mutation was distributed across Malaysia with frequency ranging from 20% in Kota Bharu to 39% in Penang (Figure 6A). The genotyping of the V1016G mutation between resistant and susceptible mosquitoes for permethrin, deltamethrin and DDT did not detect a significant correlation between V1016G and resistance to these insecticides with low OR and P > 0.05 in all samples (Additional file 1: Table S6).

##### Assessment of an additive resistance between 1534C and 1016G resistant alleles

A haplotypic association analysis was performed to assess whether there was an increased likelihood for a mosquito to become resistant when harbouring both resistant alleles rather than just one. Both resistant alleles occurred independently from one another as mosquitoes had either the 1534C allele or the 1016G allele while others had both (Additional file 1: Table S7). Only 1.1% of any homozygote resistant mosquitoes were double homozygotes resistant (RR/RR; CC/GG) to both mutations (2 out 180 mosquitoes) suggesting the possible presence of a fitness cost for this haplotype. However, an additive effect for resistance was observed for deltamethrin in Penang when comparing the double heterozygote RS/RS haplotype (F/C1534/1016 V/G) to RR/SS (C/C1534/1016 V/V) (OR = 1.63; P < 0.05) and to SS/RS (F/F1534/1016 V/G) (OR = 5.5; P < 0.001). A higher additive effect was observed when comparing the RR/RS to other haplotypes such as RS/RS (OR = 4.1; P < 0.001), SS/RS (OR = 22.4; P < 0.001) and RR/SS (OR = 6.7; P < 0.001) (Figure 6B). The additive effect was also observed for permethrin although the OR was infinite due to the absence of the RS/RS haplotype in susceptible mosquitoes. In Johor Bharu and Kota Bharu, an additive effect was also observed for deltamethrin (Table 3).

**Table 3:** **Odds ratios for the association of F1534C/V1016G haplotypes with pyrethroid resistance**

	**Penang**		**Johor Bharu**		**Kota Bharu**	
	**Perm**	**Delta**	**Perm**	**Delta**	**Perm**	**Delta**
FC/VG vs CC/VV	infinity	1.63*	0.47^ns^	2.8*	1.48^ns^	5.2***
FC/VG vs FF/VG	infinity	5.5***	0^ns^	/	0.25^ns^	0.38^ns^
CC/VG vs FC/VG	/	4.1***	infinity	0.4^ns^	/	/
CC/VG vs FF/VG	/	22.4***	infinity	/	/	/
CC/VG vs CC/VV	/	6.67***	infinity	1.1^ns^	/	/

C and G are the resistant alleles while F and V are the susceptible alleles; Perm, permethrin; Delta, deltamethrin; ns, non significant; *P < 0.05; ***P < 0.001; other genotype combinations were not compared between of insufficient number.

#### Detection of *kdr* mutations in *Ae. albopictus*

The attempt to use the same pyrosequencing assays as in *Ae. aegypti* to genotype the three codons (1011, 1016 and 1534) associated with *kdr* mutations was unsuccessful for *Ae. albopictus* samples as no pyrosequencing peak was detected despite good PCR amplifications. Therefore, the presence of potential *kdr* mutations in *Ae. albopictus* was further investigated by sequencing the cDNA fragment spanning exons 19 to 31. A 2586 bp PCR product was successfully amplified in three pools of ten DDT resistant mosquitoes from each location. Due to the presence of alternative splicing, the direct sequencing and alignment generated two fragments. The first fragment (Frag-1) covered a size of 504 bp from codon 919 to 1085. The second fragment (Frag-2), of a size of 1099 bp covered codons 1339 to 1704 of the gene.

##### Polymorphism analysis

Frag-1 (n = 18) exhibited 7 substitutions across Malaysia with a total of 8 haplotypes. No amino acid change was recorded suggesting that the 1011 and the 1016 *kdr* mutations observed in *Ae. aegypti* are absent in *Ae. albopictus* in Malaysia. A total of 13 substitutions were recorded for Frag-2 with a total of 14 haplotypes. Again, no amino acid change was observed in these samples suggesting that the F1534C mutation observed in *Ae. aegypti* and recently reported in *Ae. albopictus* in Singapore [18] is absent in these populations.

Analysis of the maximum likelihood phylogenetic tree of the haplotypes for both fragments indicated that contrary to *Ae. aegypti*, the VGSC gene exhibits a higher genetic diversity in *Ae. albopictus* (Additional file 2: Figure S4). The lack of a predominant haplotypes suggests that the VGSC gene is not under selection pressure and support the absence of *kdr* mutation in this species in Malaysia.

## Discussion

This study has mapped the distribution of resistance to the main insecticides in the two dengue vectors *Ae. aegypti* and *Ae. albopictus* across Malaysia and investigated the role of target site mutations in conferring pyrethroid resistance in both species. Overall, this study has highlighted a significant contrast between the two species in term of their resistance profiles and also the contribution of the knockdown resistance mechanism.

### Contrasting resistance profiles between *Ae. aegypti* and *Ae. albopictus* across Malaysia

The two species significantly differ in their resistance profile to pyrethroids with consistently higher prevalences of resistance observed in *Ae. aegypti* whereas *Ae. albopictus* populations are mostly fully susceptible. *Ae. albopictus* susceptibility to pyrethroids in Malaysia is in line with previous studies reporting a relative susceptibility of this species to pyrethroids across the world [31]. However, the moderate resistance observed in the Kuala Lumpur population to both permethrin and deltamethrin indicates that such resistance may be building up and calls for regular monitoring. The difference of susceptibility between the two species could be due to the fact that *Ae. albopictus,* being a more rural vector, is under less selection pressure than *Ae. aegypti* which is more confined to urban settings with higher exposure to insecticide either during fogging by the MoH [7,32] and to household insecticide exposure [33].

The contrast between the two species is also further highlighted by their resistance profiles to both malathion and dieldrin with higher proportion of resistance individuals in *Ae. albopictus* than *Aedes aegypti.* The higher dieldrin resistance in *Ae. albopictus* could be due to the ecology of *Ae. albopictus* with breeding sites near vegetation in agricultural settings where they may have been exposed to dieldrin when this insecticide was still used in agriculture for the control of soil insects [34].

However, both species also present some similarities notably regarding the widespread distribution of DDT resistance across Malaysian populations with Kuala Lumpur populations consistently more resistant as also observed for pyrethroids. High DDT resistance in both species is commonly reported across the world [31]. The widespread resistance to DDT is most likely due to the past usage of this insecticide to control *Ae. aegypti* in Malaysia [9]. The full recovery of DDT susceptibility observed for *Ae. albopictus* suggests that cytochrome P450 genes may be playing a role as observed in other mosquitoes such as in *An. gambiae* where the *CYP6M2* gene has been shown to metabolise DDT [35]. Another similarity was the low resistance level to temephos in both species despite the widespread use of this insecticide in Malaysia since the 1970s and in 1998 during the worldwide pandemic [32]. This low resistance to temephos is comparable to the susceptibility reported in another *Ae. albopictus* population from Selangor region in Malaysia [36] suggesting that larviciding with temephos probably remains efficient across Malaysia*.* However, because higher resistance levels to temephos have been observed in larvae of both species in other countries in the region such as in Thailand [37-40], resistance to this insecticide should continuously be monitored. The high level of resistance observed in Malaysia notably for *Ae. aegypti* even when using the higher *Anopheles* diagnostic doses suggests that current recommended *Aedes* diagnostic doses [29] are most likely too low for these species and should be revised. Future work using a dose–response assay based on LT50 or LD50 could help to better assess the resistance level of these populations.

### Significant role of knockdown resistance in *Ae. aegypti* contrasts to its absence in *Ae. albopictus*

#### Kdr mutations contribute to resistance in *Ae. aegypti*

The detection of the F1534C and the V1016G mutations is the first report of *kdr* resistance in Malaysian populations of *Ae. aegypti.* The detection of both mutations was also recently reported across neighboring Thailand [40]. In some locations such as in Penang, a significant correlation was established between F1534C genotypes and pyrethroid resistance revealing that the F1534C mutation significantly contributes to pyrethroid resistance. This is similar to previous findings in other strains of this species [13,31]. However, in contrast to other studies which only found a correlation between F1534C and type I pyrethroids [13,40,41], F1534C was also associated with type II pyrethroids in Penang probably because of the additive contribution of the V1016G mutation. Indeed, it was observed in Penang that the presence of the 1016G allele always increases the likelihood of various 1534 genotypes to be resistant to deltamethrin suggesting that mosquito's haplotype for both mutations is more important in determining the phenotypes than the genotype at a single mutation. However, such correlation between F1534C and pyrethroid resistance was not observed in other locations such as in Kota Bharu suggesting that presence of the F1534C mutation alone does not automatically result to resistance to pyrethroids or that other mechanisms such as metabolic resistance are playing a more predominant role in the resistance observed in such locations. Correlation of the F1534C with DDT resistance could not be properly assessed in most of the locations because of the low number of susceptible mosquitoes. However in Kota Bharu where such assessment was possible, the correlation was not significant although a trend was present (OR = 2.65, P = 0.078)].

The reduced genetic diversity observed for the VGSC fragment spanning the F1534C mutation is evidence that this gene is under selection across Malaysia. The predominance of a resistant haplotype further supports that this mutation was playing a significant role in permethrin resistance across Malaysia. This reduced diversity around this VGSC fragment is similar to cases observed around the L1014F mutation in *An. gambiae* where a major resistant haplotype was detected in West and Central Africa [42].

The detection of the V1016G mutation across Malaysia suggested that knockdown resistance is not solely explained by the F1534C although the lower frequency of the 1016G resistant allele and the lack of direct correlation with resistance suggest it is perhaps less important than the 1534C allele. The lower frequency of the 1016G allele may suggest a more recent occurrence of this mutation in Malaysia possibly through migration from neighboring countries. Indeed, the 1016G allele has previously been detected in other countries in the region such as in an *Ae. aegypti* strain from Indonesia [12], in Taiwan [43] and in Thailand [40]. However, the lack of significant correlation between the 1016G allele and resistance phenotypes in Malaysia contrasts to Thailand where such a correlation was recently established with deltamethrin resistance [40]. Nevertheless, the role of the V1016G mutation in Malaysia was revealed by the increased resistance that its presence confers to mosquitoes already possessing the 1534C allele. This additive effect of possessing two *kdr* mutations is similar to the case in the malaria vector *An. gambiae* where the N1575Y mutation increases resistance in the presence of the L1014F allele [44]. The very low frequency of double homozygote resistant mosquitoes observed in this study could suggest that there is a fitness cost associated with such haplotype as also suggested in Thailand [40]. Additionally, because a duplication of the VGSC gene was recently suggested in *Ae. aegypti* [45], it will be interesting to establish if the two *kdr* mutations detected in Malaysian *Ae. aegypti* are found on the same haplotype or not.

#### Absence of *kdr* mutation in *Ae albopitus*

The absence of *kdr* mutation in *Ae. albopictus* in Malaysia is in line with previous studies which could not detect such mutations in other populations worldwide [31,46,47]. Furthermore, the high genetic diversity observed for the two VGSC fragments and a complete lack of correlation between haplotypes and resistance phenotypes suggest that no *kdr* mutation is present in *Ae. albopictus*. This absence of *kdr* mutation is similar to the situation observed in the malaria vector *An. funestus* where the *kdr* mutation is absent despite DDT and pyrethroid resistance [48,49]. However, the first report of the detection of a *kdr* mutation in Singapore in 2011 [18] shows that such mutation should continually be monitored in *Ae. albopictus* populations in Malaysia.

The absence of *kdr* mutation in *Ae. albopictus* in contrast to *Ae. aegypti* is in line with the significant differences observed in their resistance profiles across Malaysia. Such difference suggests that both species have developed different resistance mechanisms in response to the selection pressure they face in their specific ecological niche.

#### Metabolic resistance contributes to resistance in both species

The significant recovery of susceptibility after exposure to PBO in both *Aedes* species across Malaysia suggests that metabolic resistance mechanisms are playing a significant role in the observed resistance. The synergist action of PBO particularly indicates that elevated expression of cytochrome P450 genes or other oxidase enzymes [50] is playing a major role in the various resistances observed. Such role of metabolic resistance mechanisms is in line with previous studies revealing that over-transcription of several gene families were associated with resistance to pyrethroids, DDT and organophosphates in various populations of *Ae. aegypti* [38,51,52]. With the absence of *kdr* mutations in *Ae. albopictus* it is likely that the contribution of such metabolic resistance mechanism is even greater in this species. Future genome-wide transcription studies will help to characterise these metabolic resistance mechanisms in both species across Malaysia.

## Conclusion

By characterising the frequency, geographical distribution and mechanisms of resistance to insecticides in the two major dengue vectors in Malaysia, this study has provided suitable information for the design and implementation of successful resistance management strategies against both species. Indeed, the detection of specific pyrethroid resistance mutations and the molecular diagnostic tools designed can help to track and map the spread of resistance but also to assess the response of mosquito populations to future insecticide-based interventions. Differences in the resistance profiles and mechanisms between the two species as well as between locations also highlight the need of tailoring vector control interventions to each species and to each region to increase the success of dengue control in Malaysia.

## Additional files

Additional file 1: Table S1. Primers used for amplification of the VGSC gene. **Table S2.** Primers used for pyrosequencing. **Table S3.** Adult bioassay for different strains of *Ae. aegypti* exposed to six insecticides and PBO synergist. **Table S4.** Adult bioassay for different strains of *Ae. albopictus* exposed to six insecticides and PBO synergist. **Table S5.** Polymorphism parameters of the VGSC fragment between permethrin resistant and susceptible *Ae. aegypti* across Malaysia. **Table S6.** Association of *kdr* V1016G allele count in all field populations with specific insecticide resistance phenotype. **Table S7.** Haplotype distribution for the F1534C and V1016G mutations between resistant and susceptible *Ae. aegypti* for permethrin (Perm) and deltamethrin (Delta) across Malaysia. Additional file 2: Figure S1. Detection of the V1016G mutation. (A) shows sequence chromatographs with mutation at position 1016 . (B) shows the melting curve genotyping of results. **Figure S2.** Pyrograms resulting from *kdr* F1534C pyrosequencing assay. SNP areas of interest are coloured yellow and peaks represent nucleotides conferring *kdr* genotype: T/T (homozygous susceptible), G/T (heterozygous), G/G (homozygous resistant). **Figure S3.** Correlation between the F1534C alleles and pyrethroid resistance phenotypes. (A) and (B) are for permethrin and Deltamethrin in Penang respectively whereas (C) and (D) are for the same insecticide in Johor Bharu respectively. **Figure S4.** Maximum likelihood phylogenetic tree of VGSC haplotypes in *Ae. albopictus* after cDNA sequencing.
