# Supplementary material for: Contrasting patterns of insecticide resistance and knockdown resistance (kdr) in the dengue vectors Aedes aegypti and Aedes albopictus from Malaysia
Source: Parasit Vectors. 2015 Mar 25;8:181. doi: 10.1186/s13071-015-0797-2 (PMC4377062; doi:10.1186/s13071-015-0797-2)
Supplement: Additional file 1: Table S1. — Primers used for amplification of the VGSC gene. Table S2. Primers used for pyrosequencing. Table S3. Adult bioassay for different strains of Ae. aegypti exposed to six insecticides and PBO synergist. Table S4. Adult bioassay for different strains of Ae. albopictus exposed to six insecticides and PBO synergist. Table S5. Polymorphism parameters of the VGSC fragment between permethrin resistant and susceptible Ae. aegypti across Malaysia. Table S6. Association of kdr V1016G allele count in all field populations with specific insecticide resistance phenotype. Table S7. Haplotype distribution for the F1534C and V1016G mutations between resistant and susceptible Ae. aegypti for permethrin (Perm) and deltamethrin (Delta) across Malaysia. [file 13071_2015_797_MOESM1_ESM.docx]

**Table S1:** Primers used for amplification of the VGSC gene

| **Primer Name** | **Sequence** | **Product size** |
| --- | --- | --- |
| In26ex29 F | TCT CAT CTC TCC GAA GAT GCT CTG TA | 1026 bp |
| In26ex29 R | TCC TCC GTC ATG AAC ATT TCC AGT G |  |
| cDNAex19ex31 F | CTT CGA GTG TTC AAG CTA GCG AAA TC | 2586 bp |
| cDNAex19ex31 R | CTG AAA CAG CAG GAT CAT GCT CTG |  |

**Table S2:** Primers used for pyrosequencing

| **Primer Name** | **Sequence** | **Modification** |
| --- | --- | --- |
| Ae*kdr*1016pyrF | CTTTCGTGCTAACCGACAAATT |  |
| Ae*kdr*1016pyrR-bio | AAAAGAATCGGAACGAAAACAGG | Biotinylated |
| Ae*kdr*1016pyrseq | AATTGTTTCCCACTCG |  |
| Sequence to analyse | CACAG A/G G/T ACT C/T AACCT |  |
| Ae*kdr*1011pyrF | TATGCTTGTGGGTGACGTGT |  |
| Ae*kdr*1011pyrR-bio | GCTGCTAGCACGCCTCTAAT | Biotinylated |
| Ae*kdr*1011pyrseq | TTCTTTTTGGCCACCG |  |
| Sequence to analyse | TAGTG A/G T A/G GGAAATCTAG |  |
| Ae*kdr*1534pyrF | TCGCGGGAGGTAAGTTATTG |  |
| Ae*kdr*1534pyrR-bio | CGATGATGACACCGATGAAC | Biotinylated |
| Ae*kdr*1534pyrseq | TACTTTGTGTTCTTCA |  |
| Sequence to analyse | TCATCT G/T CGGGTCGT |  |

Highlighted nucleotides show position of mutation.

**Table S3:** Adult bioassay for different strains of *Ae. aegypti* exposed to six insecticides and PBO synergist

| **INSECTICIDE** | **% MORTALITY ± SD** | | | | | | | |
| --- | --- | --- | --- | --- | --- | --- | --- | --- |
|  | **PENANG** | | **KUALA LUMPUR** | | **JOHOR BHARU** | | **KOTA BHARU** | |
|  | Male | Female | Male | Female | Male | Female | Male | Female |
| PERMETHRIN | 43 ± 1.71 (100) | 33 ± 1  (100) | 0  (100) | 1 ± 0.5  (100) | 72 ± 3.6 (100) | 59.2 ± 1.3 (125) | 8 ± 1.6  (100) | 10 ± 2.08 (100) |
| DELTAMETHRIN | 77 ± 1.5 (100) | 72 ± 2.94 (100) | 9 ± 2.6  (100) | 0  (100) | 100 ± 0  (100) | 79 ± 1.7 (100) | 91 ± 2.6 (100) | 82 ± 1.3 (100) |
| DDT | 50 ± 2.38 (100) | 17 ± 1  (100) | 0  (100) | 0  (100) | 57 ± 3.5 (100) | 50.4 ± 1.5 (125) | 8 ± 2.2  (100) | 10 ± 2.1 (100) |
| BENDIOCARB | 27 ± 1.5 (100) | 53 ± 1.26 (100) | 20 ± 3.6 (100) | 44 ± 3.6 (100) | 38.4 ± 3.7 (125) | 25 ± 1.7 (100) | 84 ± 3.7 (100) | 91 ± 1  (100) |
| MALATHION | 100 ± 0  (100) | 100 ± 0  (100) | 100 ± 0  (100) | 91 ± 2.2 (100) | 98 ± 1  (100) | 99 ± 0.5 (100) | 100 ± 0  (100) | 100 ± 0  (100) |
| DIELDRIN | 100 ± 0  (100) | 100 ± 0  (100) | 100 ± 0  (100) | 100 ± 0  (100) | 84 ± 0.8 (100) | 88 ± 2  (100) | 100 ± 0  (100) | 100 ± 0  (100) |
| PBO - PERMETHRIN | 100 ± 0  (100) | 100 ± 0  (100) | 93 ± 3.5 (100) | 26 ± 1.3 (100) | 100 ± 0  (100) | 100 ± 0  (50) | * | 5 ± 0  (25) |
| PBO - DELTAMETHRIN | 100 ± 0  (100) | 100 ± 0  (100) | 87 ± 3.9 (100) | 71 ± 2.6 (100) | 100 ± 0  (100) | 100 ± 0  (50) | * | * |
| PBO - DDT | 100 ± 0  (100) | 55 ± 1  (100) | 68 ± 1.4 (100) | 35 ± 1.5 (100) | 97 ± 1.5 (100) | 80 ± 5.2  (100) | * | * |
| PBO - BENDIOCARB | 95 ± 3  (100) | 65 ± 2.5  (100) | 87 ± 2.6 (100) | 98 ± 0.6 (100) | 97 ± 1  (100) | 93 ± 2.2 (100) | * | * |

In brackets are the numbers of mosquitoes tested (n). * indicates no data has been produced due to low sample size. SD: Standard deviation.

**Table S4:** Adult bioassay for different strains of *Ae. albopictus* exposed to six insecticides and PBO synergist

| **INSECTICIDE** | **% MORTALITY ± SD** | | | | | | | |
| --- | --- | --- | --- | --- | --- | --- | --- | --- |
|  | **PENANG** | | **KUALA LUMPUR** | | **JOHOR BHARU** | | **KOTA BHARU** | |
|  | Male | Female | Male | Female | Male | Female | Male | Female |
| PERMETHRIN | 95.2 ± 1.1 (125) | 100 ± 0  (125) | 81 ± 6.6 (100) | 87 ± 3.9  (100) | 100 ± 0  (50) | 100 ± 0  (50) | 100 ± 0  (100) | 100 ± 0  (100) |
| DELTAMETHRIN | 100 ± 0  (125) | 100 ± 0  (125) | 93 ± 1  (100) | 89 ± 1.7  (100) | 100 ± 0  (50) | 100 ± 0  (50) | 100 ± 0  (100) | 97 ± 1.5  (100) |
| DDT | 79.2 ± 3.3 (125) | 96.8 ± 1.1 (125) | 17 ± 3.3 (100) | 6 ± 1  (100) | 98 ± 0.71  (50) | 60 ± 1.41  (50) | 4 ± 1  (100) | 14 ± 3.7  (100) |
| BENDIOCARB | 32 ± 1.6 (125) | 28.8 ± 1.9 (125) | 45 ± 3.5  (100) | 31 ± 4.4  (100) | 100 ± 0  (50) | 44 ± 1.41  (50) | 98.7 ± 0.6 (100) | 93 ± 1.5  (100) |
| MALATHION | 76.8 ± 2.3 (125) | 100 ± 0  (125) | 84 ± 0.6 (100) | 76 ± 0.8  (100) | 82 ± 0.71  (50) | 68 ± 0  (50) | 100 ± 0  (100) | 100 ± 0  (100) |
| DIELDRIN | 75.2 ± 3.1 (125) | 40 ± 1.4  (125) | 90 ± 1.3 (100) | 84 ± 2.58  (100) | 100 ± 0  (50) | 90 ± 0.71  (50) | 100 ± 0  (100) | 100 ± 0  (100) |
| PBO - DDT | 97 ± 1  (100) | 99 ± 0.5  (100) | 91 ± 1.7  (100) | 52 ± 2.9  (100) | * | * | * | * |
| PBO - BENDIOCARB | 79 ± 1  (100) | 53 ± 1.3  (100) | 100 ± 0  (100) | 57 ± 1.7  (100) | * | * | * | * |

In brackets are the numbers of mosquitoes tested (n). * indicates no data has been produced due to low sample size. SD: Standard deviation.

**Table S5: Polymorphism parameters of the VGSC fragment between permethrin resistant and susceptible *Ae. aegypti* across Malaysia.**

| **Entire sequence region** | | | | | | | | | **Coding region** | | | | | | | **Non-coding region** | | | | |
| --- | --- | --- | --- | --- | --- | --- | --- | --- | --- | --- | --- | --- | --- | --- | --- | --- | --- | --- | --- | --- |
| **Samples** | **N** | **S** | **h** | **Syn** | **NSyn** | **π (k)** | **D** | **D*** | **S** | **h** | **Syn** | **NSyn** | **π (k)** | **D** | **D*** | **S** | **h** | **π (k)** | **D** | **D*** |
| **Penang** | | | | | | | | | | | | | | | | | | | | |
| Alive | 10 | 3 | 2 | 0 | 2 | 0.00226 (1.84) | 1.98* | 1.15 | 1 | 2 | 0 | 1 | 0.00169 (0.73) | 0.12 | -0.28 | 2 | 2 | 0.00294 (1.11) | 1.84 | 1.03 |
| Dead | 12 | 2 | 3 | 0 | 0 | 0.00087 (0.71) | 0.22 | 0.97 | 0 | 1 | 0 | 0 | 0 | - | - | 2 | 3 | 0.00188 (0.71) | 0.22 | 0.97 |
| Total | 22 | 4 | 4 | 0 | 2 | 0.00164 (1.34) | 0.62 | 0.14 | 2 | 2 | 0 | 2 | 0.00093 (0.40) | -0.6 | -0.63 | 2 | 3 | 0.00249 (0.93) | 1.61 | 0.85 |
| **Kuala Lumpur** | | | | | | | | | | | | | | | | | | | | |
| Alive | 8 | 3 | 3 | 0 | 1 | 0.00201 (1.64) | 1.73 | 1.23 | 1 | 2 | 0 | 1 | 0.00131 (0.57) | 1.44 | 0.89 | 2 | 2 | 0.00283 (1.07) | 1.45 | 1.11 |
| Dead | 2 | 0 | 1 | 0 | 0 | 0 | - | - | 0 | 1 | 0 | 0 | 0 | - | - | 0 | 1 | 0 | - | - |
| Total | 10 | 3 | 3 | 0 | 1 | 0.00202 (1.64) | 1.98* | 1.15 | 1 | 2 | 0 | 1 | 0.00123 (0.53) | 1.3 | 0.8 | 2 | 2 | 0.00294 (1.11) | 1.84 | 1.03 |
| **Johor Bharu** | | | | | | | | | | | | | | | | | | | | |
| Alive | 10 | 3 | 3 | 0 | 1 | 0.00106 (0.87) | -0.66 | -0.8 | 1 | 2 | 0 | 1 | 0.00046 (0.2) | -1.12 | -1.24 | 2 | 3 | 0.00176 (0.67) | -0.18 | -0.28 |
| Dead | 10 | 1 | 2 | 0 | 0 | 0.00065 (0.53) | 1.3 | 0.8 | 0 | 1 | 0 | 0 | 0 | - | - | 1 | 2 | 0.00141 (0.53) | 1.3 | 0.8 |
| Total | 20 | 3 | 3 | 0 | 1 | 0.00083 (0.68) | -0.53 | -1.25 | 1 | 2 | 0 | 1 | 0.00023 (0.1) | -1.16 | -1.54 | 2 | 3 | 0.00153 (0.58) | 0.06 | -0.59 |
| **Kota Bharu** | | | | | | | | | | | | | | | | | | | | |
| Alive |  |  |  |  |  |  |  |  |  |  |  |  |  |  |  |  |  |  |  |  |
| Dead | 10 | 3 | 2 | 0 | 1 | 0.00131 (1.07) | 0.02 | 1.15 | 1 | 2 | 0 | 1 | 0.00082 (0.36) | 0.01 | 0.8 | 2 | 2 | 0.00188 (0.71) | 0.02 | 1.03 |
| Total |  |  |  |  |  |  |  |  |  |  |  |  |  |  |  |  |  |  |  |  |
| **Combined** | | | | | | | | | | | | | | | | | | | | |
| Alive | 28 | 3 | 4 | 0 | 2 | 0.00193 (1.58) | 2.28* | 0.52 | 2 | 3 | 0 | 1 | 0.00130 (0.57) | 0.21 | -0.71 | 2 | 3 | 0.00267 (1.01) | 2.02* | 0.82 |
| Dead | 34 | 3 | 5 | 0 | 1 | 0.00181 (1.48) | 2.30* | 0.93 | 1 | 2 | 0 | 1 | 0.00115 (-0.50) | 1.56 | 0.58 | 2 | 4 | 0.0026 (0.98) | 1.97 | 0.79 |
| Total | 62 | 3 | 6 | 0 | 2 | 0.00184 (1.50) | 2.555 | -0.15 | 2 | 3 | 0 | 1 | 0.00120 (0.52) | 0.38 | -0.95 | 2 | 4 |  |  |  |

N= number of sequences (2n); S, number of polymorphic sites; h, Number of haplotypes; (haplotype diversity); Syn, synonymous; Nsyn, non-synonymous; π, nucleotide diversity (k= mean number of nucleotide differences); Tajima’s D and Fu and Li’s D* statistics (* P<0.05).

**Table S6: Association of *kdr* V1016G allele count in all field populations with specific insecticide resistance phenotype.**

| **Population** | **Insecticide** | **Phenotype** | **n** | **V1016G alleles** | | **Odds ratio** | **P value** |
| --- | --- | --- | --- | --- | --- | --- | --- |
|  |  |  |  | **GTA** | **GGA** |  |  |
| Penang | Perm | R | 25 | 25 | 25 | 1.2857 | 0.6629 |
|  |  | S | 8 | 9 | 7 |  |  |
|  | Delta | R | 25 | 28 | 22 | 1.1786 | 0.6892 |
|  |  | S | 25 | 30 | 20 |  |  |
|  | DDT | R | 25 | 28 | 22 | * | * |
|  |  | S | 0 | 0 | 0 |  |  |
| Kuala Lumpur | Perm | R | 25 | 20 | 30 | * | * |
|  |  | S | 1 | 2 | 0 |  |  |
|  | Delta | R | 25 | 24 | 26 | * | * |
|  |  | S | 0 | 0 | 0 |  |  |
|  | DDT | R | 25 | 22 | 28 | * | * |
|  |  | S | 0 | 0 | 0 |  |  |
| Johor Bharu | Perm | R | 25 | 45 | 5 | 0.0111 | <0.0001 |
|  |  | S | 11 | 2 | 20 |  |  |
|  | Delta | R | 25 | 42 | 8 | 1.1156 | 0.8414 |
|  |  | S | 24 | 41 | 7 |  |  |
|  | DDT | R | 25 | 48 | 2 | 0.0139 | 0.0016 |
|  |  | S | 2 | 1 | 3 |  |  |
| Kota Bharu | Perm | R | 25 | 42 | 8 | 0.3537 | 0.0794 |
|  |  | S | 10 | 13 | 7 |  |  |
|  | Delta | R | 18 | 23 | 13 | 2.004 | 0.1502 |
|  |  | S | 25 | 39 | 11 |  |  |
|  | DDT | R | 25 | 39 | 11 | 0.3447 | 0.0544 |
|  |  | S | 10 | 11 | 9 |  |  |

n = number of samples tested. For some samples, odds ratio and p value could not be calculated.

**Table S7:** Haplotype distribution for the F1534C and V1016G mutations between resistant and susceptible *Ae. aegypti* for permethrin (Perm) and deltamethrin (Delta) across Malaysia

|  | **Penang** | | | | **Johor Bharu** | | | | **Kota Bharu** | | | | **Kuala Lumpur** | | | | | **Total Malaysia** | | | |
| --- | --- | --- | --- | --- | --- | --- | --- | --- | --- | --- | --- | --- | --- | --- | --- | --- | --- | --- | --- | --- | --- |
|  | **Perm** | | **Delta** | | **Perm** | | **Delta** | | **Perm** | | **Delta** | | **Perm** | | **Delta** | | | **Perm** | | **Delta** | |
|  | **S** | **R** | **S** | **R** | **S** | **R** | **S** | **R** | **S** | **R** | **S** | **R** | **S** | **R** | **S** | **R** |  | **S** | **R** | **S** | **R** |
| SS/SS FF/VV | 0 | 0 | 0 | 0 | 0 | 0 | 1 | 0 | 0 | 6 | 0 | 0 | 0 | 0 | 0 | 0 |  | 0 | 6 | 1 | 0 |
| SS/RS FF/VG | 7 | 6 | 9 | 2 | 0 | 1 | 0 | 0 | 1 | 4 | 1 | 3 | 0 | 0 | 0 | 0 |  | 8 | 11 | 10 | 5 |
| SS/RR FF/GG | 0 | 3 | 0 | 0 | 0 | 0 | 0 | 0 | 1 | 0 | 2 | 1 | 0 | 2 | 0 | 1 |  | 1 | 7 | 2 | 3 |
| RR/SS CC/VV | 0 | 2 | 4 | 3 | 9 | 19 | 16 | 15 | 3 | 2 | 13 | 3 | 0 | 1 | 0 | 1 |  | 12 | 25 | 33 | 23 |
| RR/RS CC/VG | 0 | 0 | 1 | 5 | 0 | 2 | 3 | 3 | 0 | 0 | 0 | 0 | 0 | 0 | 0 | 0 |  | 0 | 2 | 4 | 8 |
| RR/RR CC/GG | 0 | 0 | 0 | 1 | 0 | 0 | 0 | 0 | 0 | 0 | 0 | 0 | 0 | 0 | 0 | 0 |  | 0 | 0 | 0 | 1 |
| RS/SS FC/VV | 1 | 1 | 2 | 1 | 0 | 1 | 1 | 2 | 0 | 7 | 3 | 3 | 0 | 1 | 0 | 2 |  | 1 | 11 | 6 | 10 |
| RS/RS FC/VG | 0 | 13 | 9 | 11 | 2 | 2 | 2 | 5 | 4 | 4 | 6 | 7 | 0 | 16 | 0 | 18 |  | 6 | 51 | 17 | 59 |
| RS/RR FC/GG | 0 | 0 | 0 | 0 | 0 | 0 | 1 | 0 | 0 | 0 | 0 | 0 | 0 | 5 | 0 | 3 |  | 0 | 10 | 1 | 6 |

S, susceptible; R, resistant; SS/SS is F1534/F1534:V1016/V1016; RS/RS is F1534/1534C: V1016/1016G
