# Supplementary material for: Contrasting patterns of insecticide resistance and knockdown resistance (kdr) in the dengue vectors Aedes aegypti and Aedes albopictus from Malaysia
Source: Parasit Vectors. 2015 Mar 25;8:181. doi: 10.1186/s13071-015-0797-2 (PMC4377062; doi:10.1186/s13071-015-0797-2)
Supplement: Additional file 2: Figure S1. — Detection of the V1016G mutation. (A) shows sequence chromatographs with mutation at position 1016 . (B) shows the melting curve genotyping of results. Figure S2. Pyrograms resulting from kdr F1534C pyrosequencing assay. SNP areas of interest are coloured yellow and peaks represent nucleotides conferring kdr genotype: T/T (homozygous susceptible), G/T (heterozygous), G/G (homozygous resistant). Figure S3. Correlation between the F1534C alleles and pyrethroid resistance phenotypes. (A) and (B) are for permethrin and Deltamethrin in Penang respectively whereas (C) and (D) are for the same insecticide in Johor Bharu respectively. Figure S4. Maximum likelihood phylogenetic tree of VGSC haplotypes in Ae. albopictus after cDNA sequencing. [file 13071_2015_797_MOESM2_ESM.pdf]

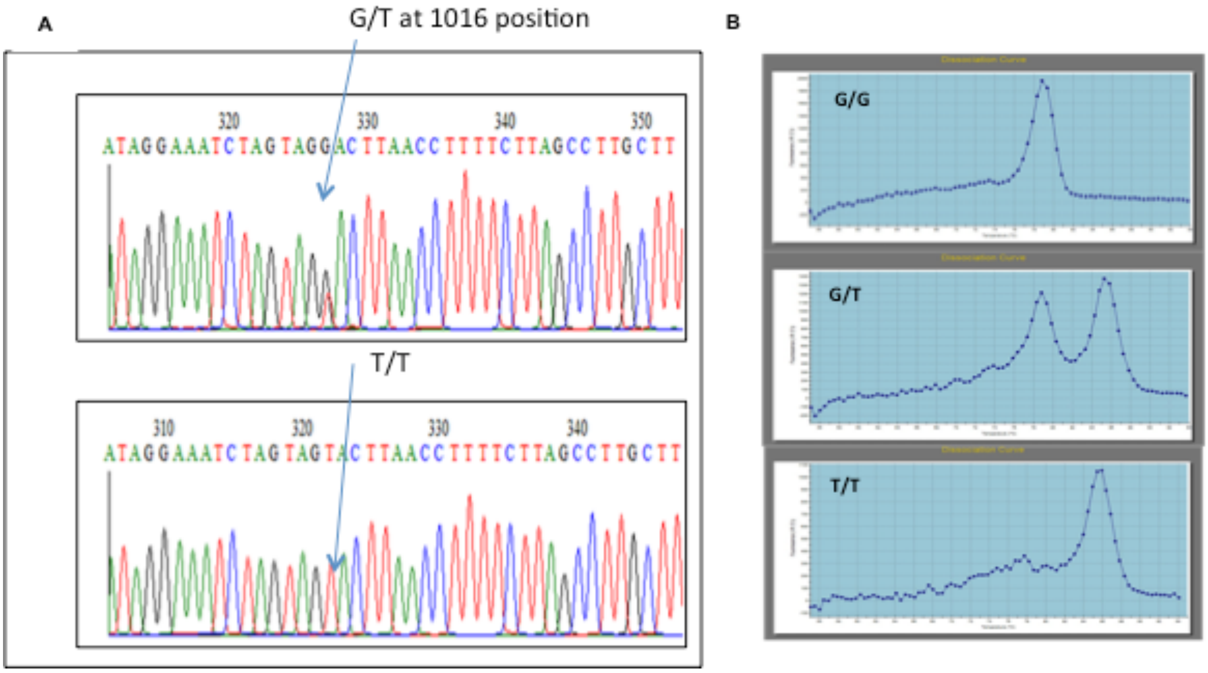

Figure S1: Detection of the V1016G mutation. (A) shows sequence chromatographs with mutation at position 1016 . (B) shows the melting curve genotyping of results.

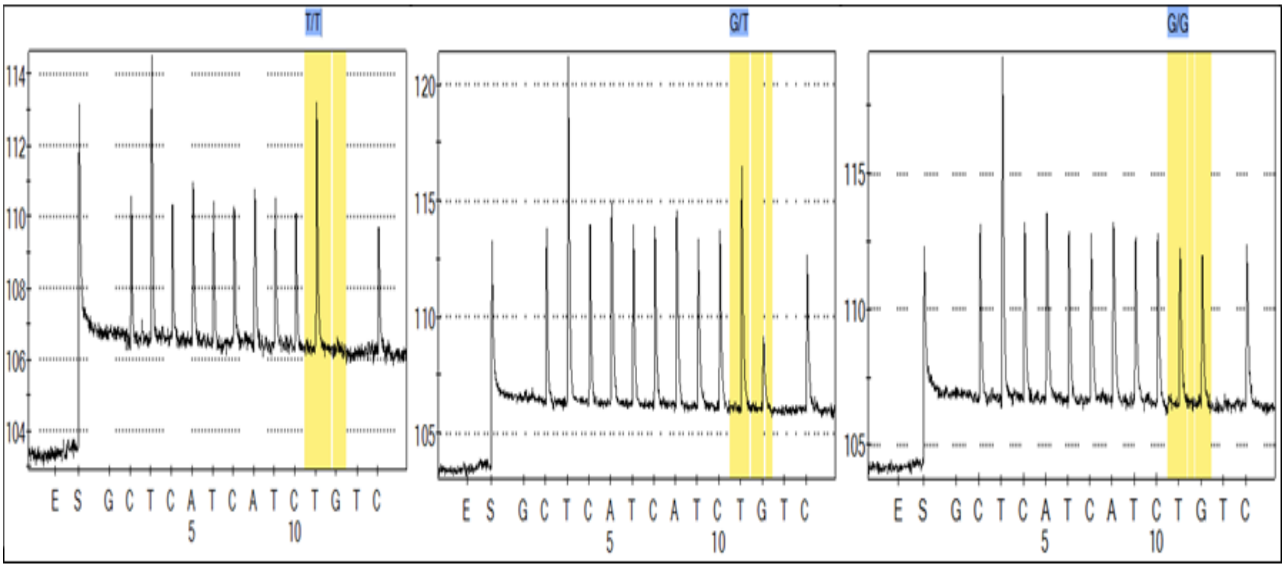

Figure S2: Pyrograms resulting from *kdr* F1534C pyrosequencing assay. SNP areas of interest are coloured yellow and peaks represent nucleotides conferring *kdr* genotype: T/T (homozygous susceptible), G/T (heterozygous), G/G (homozygous resistant).

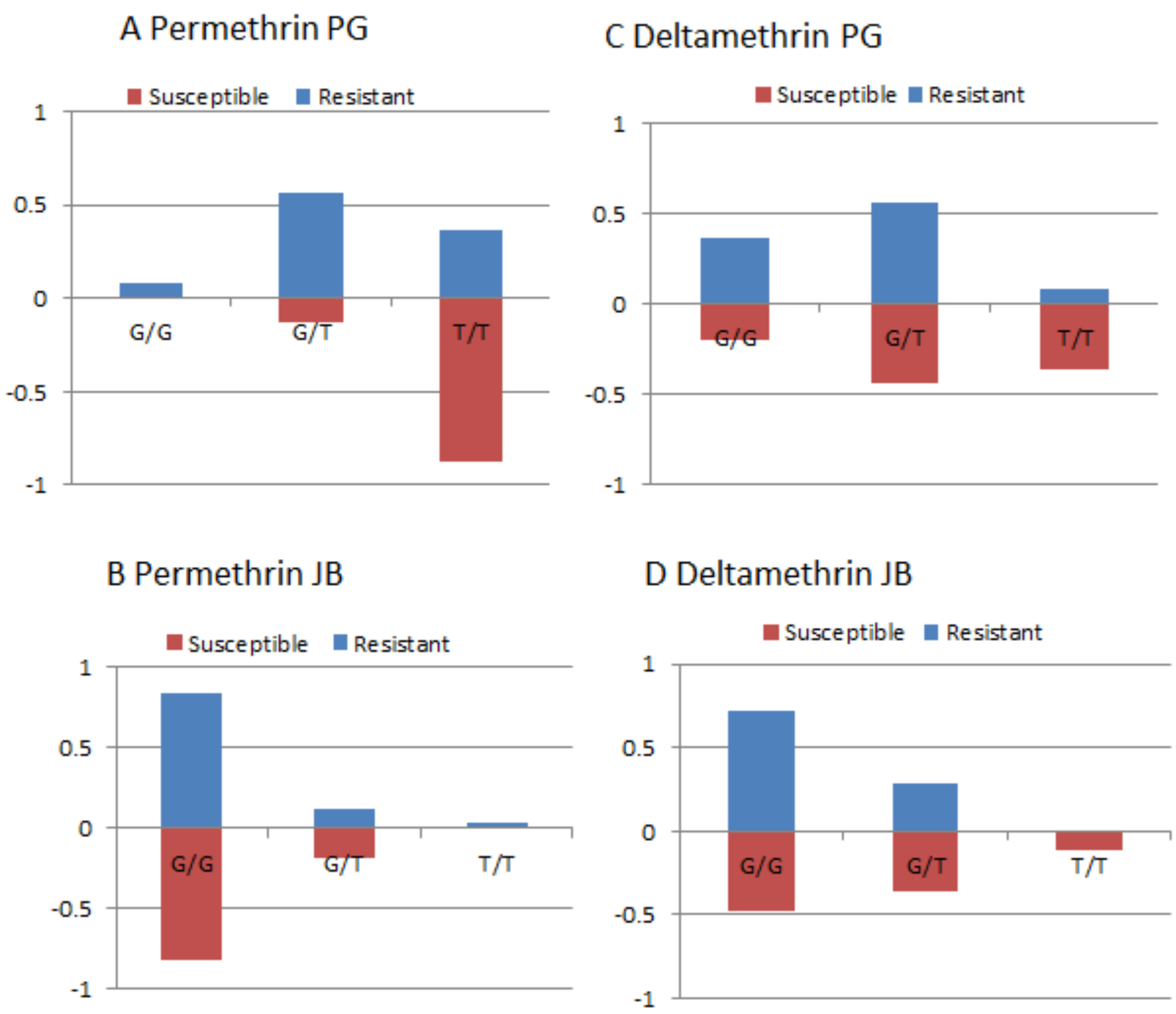

Figure S3: Correlation between the F1534C alleles and pyrethroid resistance phenotypes. (A) and (B) are for permethrin and Deltamethrin in Penang respectively whereas (C) and (D) are for the same insecticide in Johor Bharu respectively.

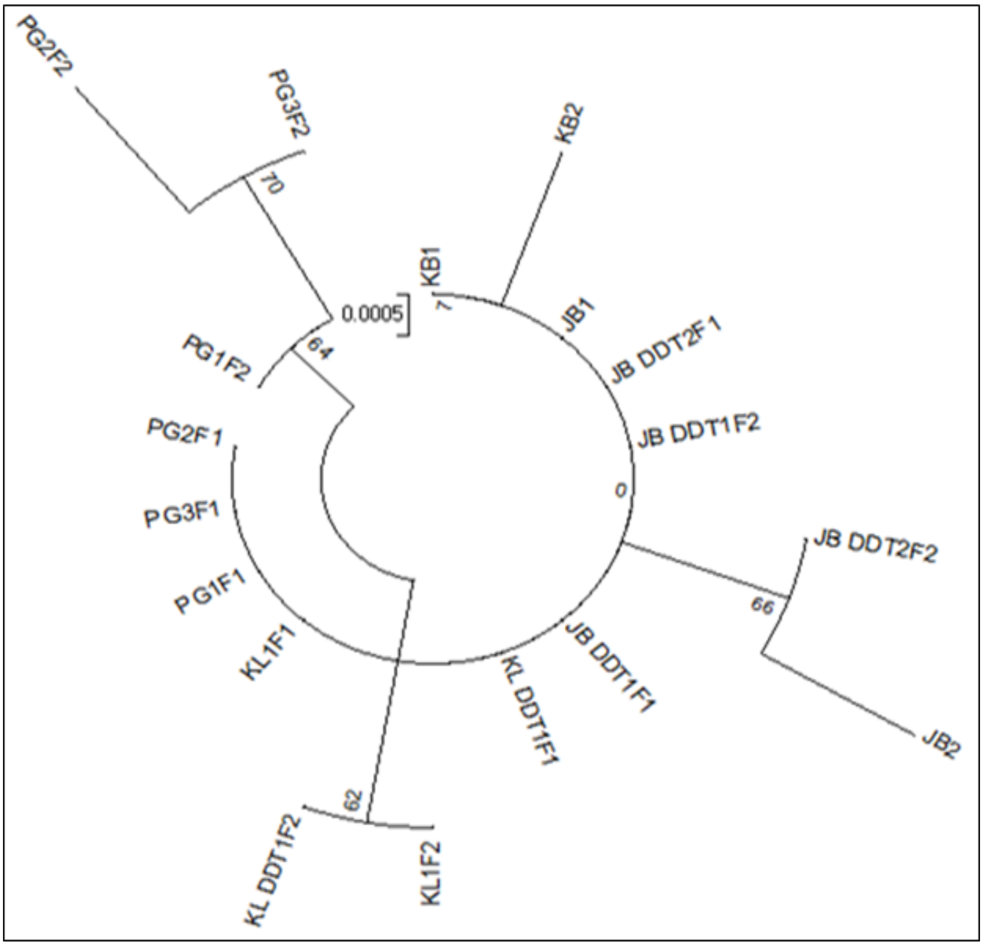

Figure S4: Maximum likelihood phylogenetic tree of VGSC haplotypes in *Ae. albopictus* after cDNA sequencing.
